# Subacute Combined Degeneration Secondary to Nitrous Oxide Toxicity

**DOI:** 10.1155/crra/9512509

**Published:** 2026-03-10

**Authors:** George Hanna, Panagiotis Gavathas, Jerome Hanna, Mina Mousa

**Affiliations:** ^1^ Department of Radiology, The University of Texas Health Science Center at San Antonio, San Antonio, Texas, USA, uthscsa.edu; ^2^ Department of Radiation Oncology, Loma Linda University Medical Center, Loma Linda, California, USA, lomalindahealth.org; ^3^ Department of Anesthesiology, The University of Texas at Tyler, Tyler, Texas, USA, uttyler.edu; ^4^ Tallahassee Memorial Healthcare INC, Radiology Associates of Tallahassee, Tallahassee, Florida, USA

**Keywords:** nitrous oxide, subacute combined degeneration, vitamin B12 deficiency

## Abstract

Subacute combined degeneration (SCD) of the spinal cord is a neurological condition caused by vitamin B12 deficiency and is most associated with cases of malabsorption, nitrous oxide exposure, or pernicious anemia. The lateral corticospinal tracts and dorsal columns are classically affected, leading to neurologic symptoms including gait disturbance, sensory deficits, and weakness. We report the case of a 35‐year‐old man who presented with paraesthesia and weakness of the upper and lower extremities bilaterally, along with urinary incontinence. Examination revealed a long segment of signal abnormality and enhancement involving the cervical and thoracic spinal cord, predominantly along the bilateral dorsal column. Upon further investigation, the patient reported a chronic history of recreational nitrous oxide abuse resulting in vitamin B12 deficiency inevitably leading to the development of SCD of the spinal cord.

## 1. Introduction

Nitrous oxide is a widely used anesthetic within the medical field, yet it is also frequently abused as an illicit recreational inhalant [1]. Prolonged use causes neurotoxicity by interfering with the bioavailability of vitamin B12, resulting in peripheral neuropathies, myelopathies, psychosis, and other neurological consequences [2]. When inhaled in high concentrations, nitrous oxide converts vitamin B12 from its active bivalent form to its inactive monovalent form, which can lead to depletion of B12 [3]. Vitamin B12 deficiency caused by chronic nitrous oxide exposure may lead to subacute combined degeneration (SCD), which is a demyelinating disorder that primarily involves the dorsal columns and lateral corticospinal tracts of the spinal cord. Patients typically present with symptoms such as gait ataxia, loss of vibration and proprioception, symmetric paresthesias, spasticity, and bladder dysfunction [4]. Imaging with magnetic resonance imaging (MRI) plays a pivotal role in diagnosis, where it typically demonstrates longitudinal T2 hyperintensity of the dorsal column [4]. Here we report a case of a patient with nitrous oxide mediated SCD diagnosed by MRI that showed abnormal signal changes on T1 and T2 with patchy postcontrast enhancement.

## 2. Case Presentation

**Figure 2:**
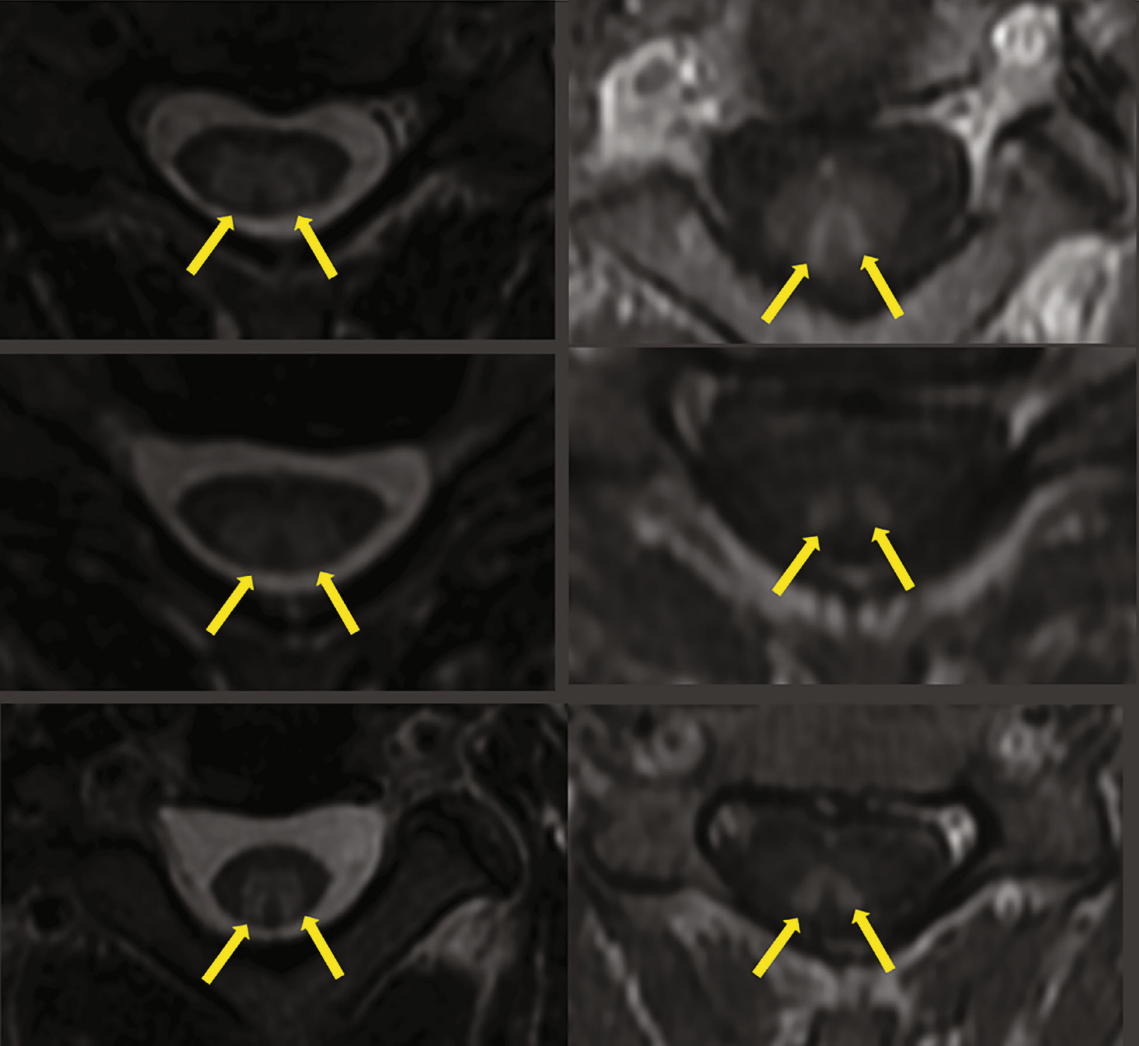
Axial T2 and axial postcontrast T1 of the cervical spine at the C2, C4, and C5 levels demonstrate T2 hyperintensity and patchy enhancement involving the dorsal columns of the cervical spinal cord (yellow arrows).

A 35‐year‐old man presented to the hospital with 6–8 weeks of progressive weakness and numbness/tingling from the neck down, including his upper and lower extremities. Physical examination was significant for gait ataxia and a positive Romberg test. Neurological exam of the patient revealed 4/5 weakness in the bilateral lower extremities that eventually progressed to complete paraplegia. The patient also reported urinary incontinence for the past 5 days. Computed tomography (CT) scan of the head/brain without contrast showed no intracranial abnormalities. CT of the cervical, thoracic, and lumbar spine was unremarkable. The patient then underwent MRI of the brain and whole spine with contrast for further evaluation of the spinal cord. Sagittal T1, T2, STIR, and postcontrast T1 images of the cervical spine demonstrate a long segment of patchy hyperintensity and enhancement within the dorsal columns (Figure 1). MRI of the cervical and thoracic spine revealed T2 hyperintense signal changes involving the cervical and upper thoracic spinal cord predominantly located to the dorsal column bilaterally with patchy enhancement extending from the C1 to the T4 levels (Figure 2). MRI of the brain and lumbar spine showed no evidence of white matter signal abnormalities or abnormal enhancement. The patient reported a chronic history of recreational nitrous oxide use for 6 months, though he was unsure of the specific amount. Further laboratory workup revealed vitamin B12 deficiency and macrocytosis. The patient was diagnosed with SCD of the spinal cord due to functional vitamin B12 deficiency secondary to nitrous oxide abuse. The patient was started on a treatment regimen consisting of vitamin B12 1000 mcg IM ×7 days, weekly ×4 weeks, then monthly injections for life. Progressive improvement of neuromuscular symptoms was noted after initiation of vitamin replacement.

Figure 1(a) Sagittal T1, (b) T2, (c) STIR, and (d) postcontrast T1 images of the cervical spine demonstrate long segment of the dorsal column T2 hyperintensity on T2‐weighted images (yellow arrows) and patchy enhancement on contrast‐enhanced T1‐weighted images (yellow arrows).

**Figure:**
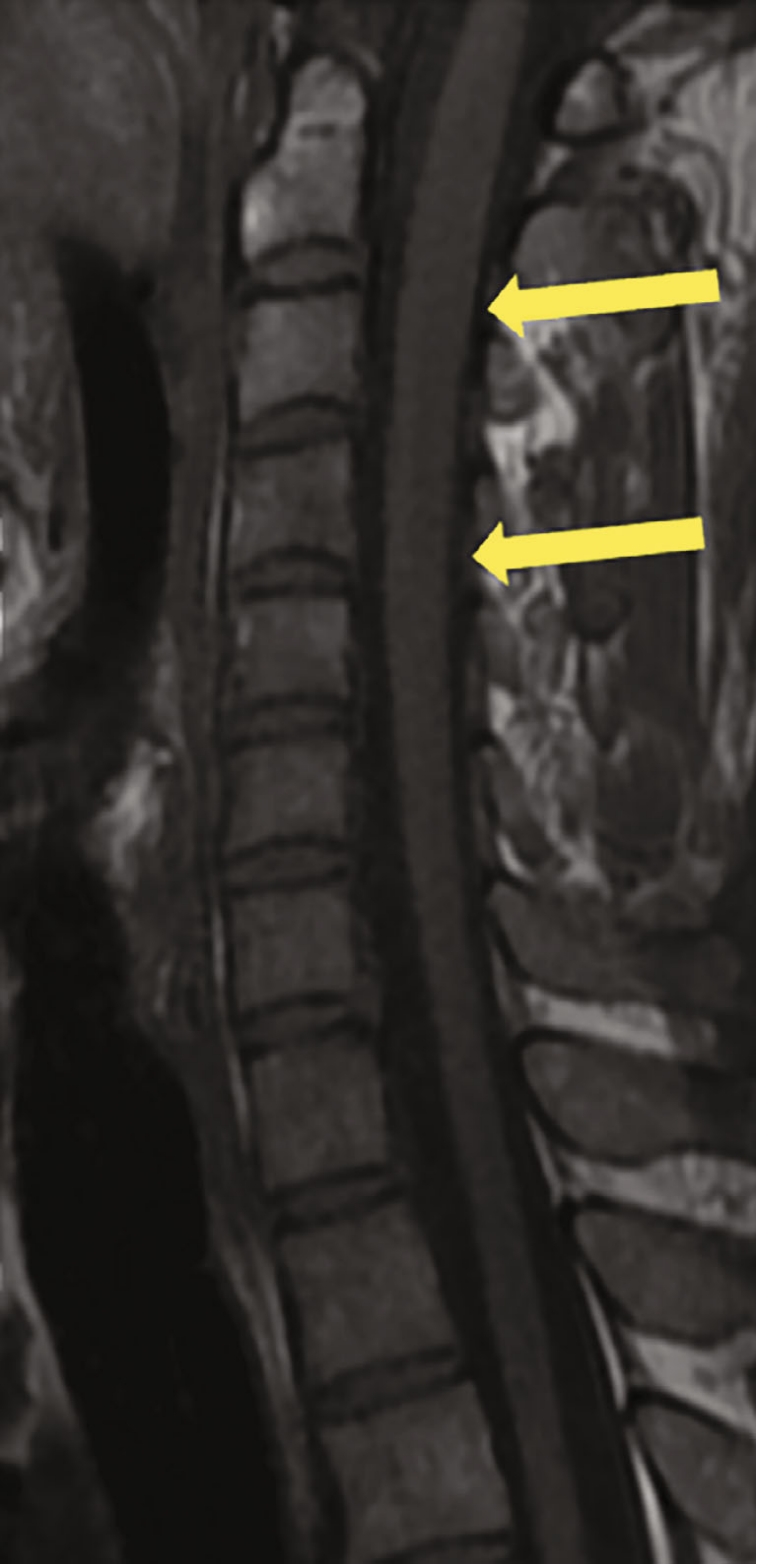
(a)

**Figure:**
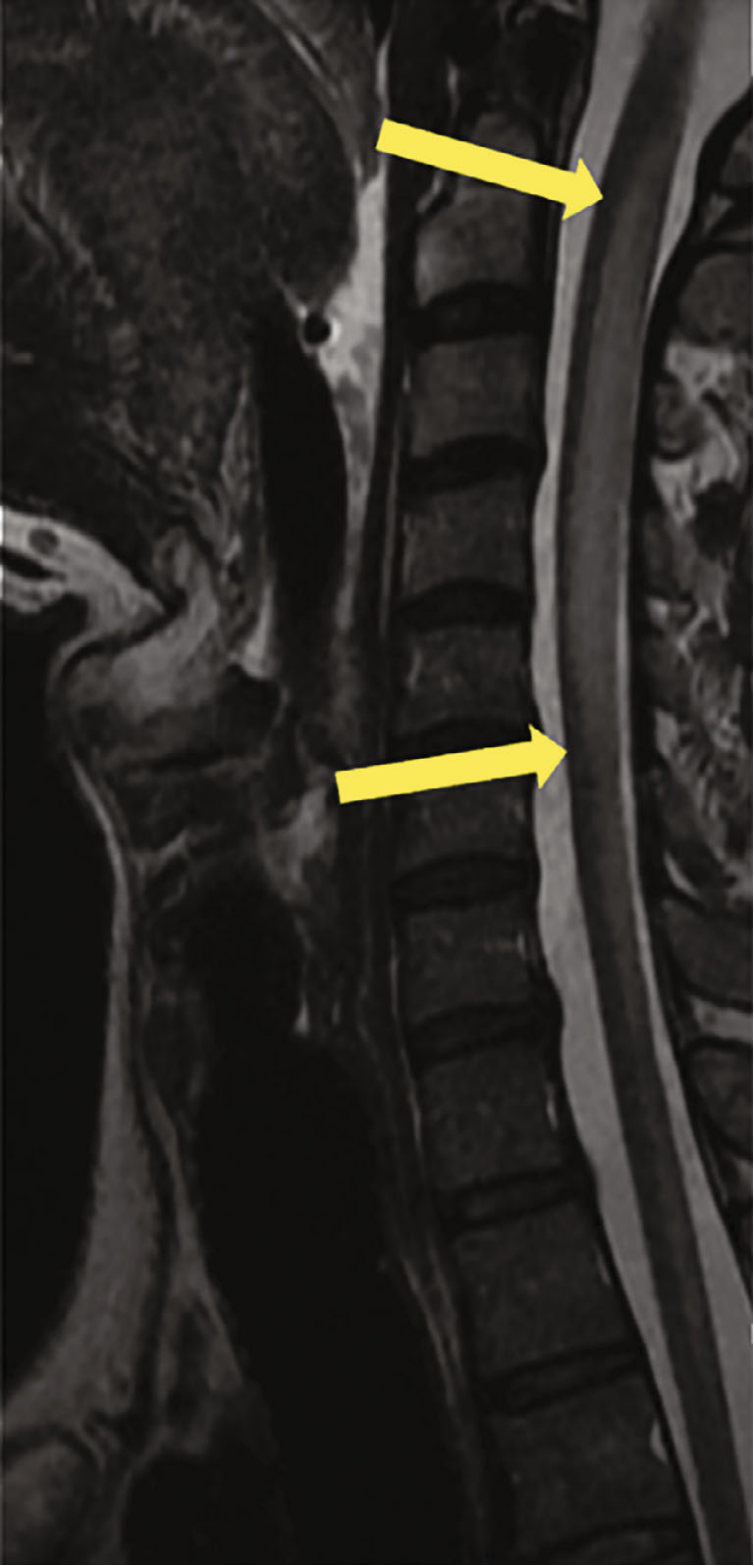
(b)

**Figure:**
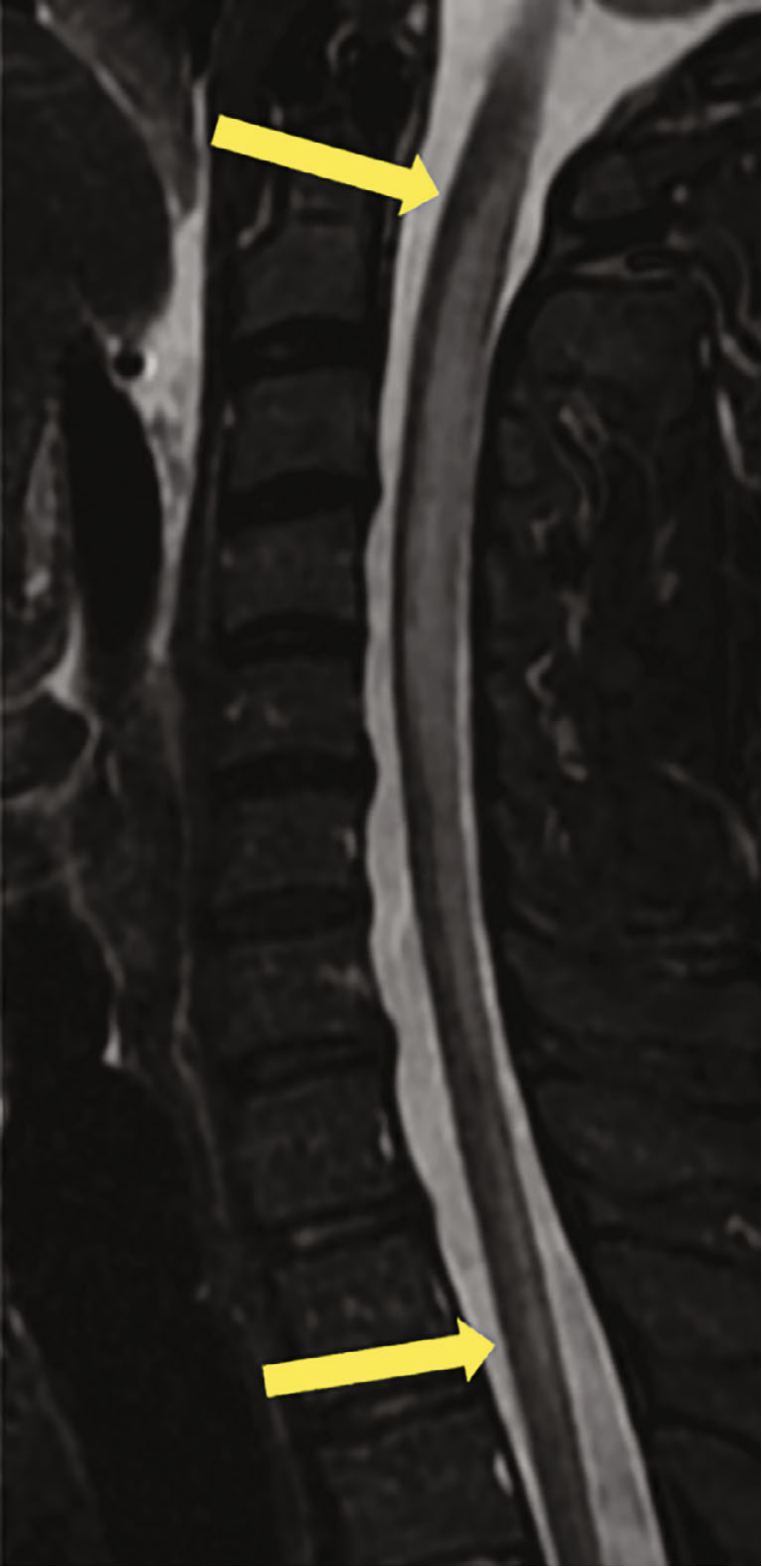
(c)

**Figure:**
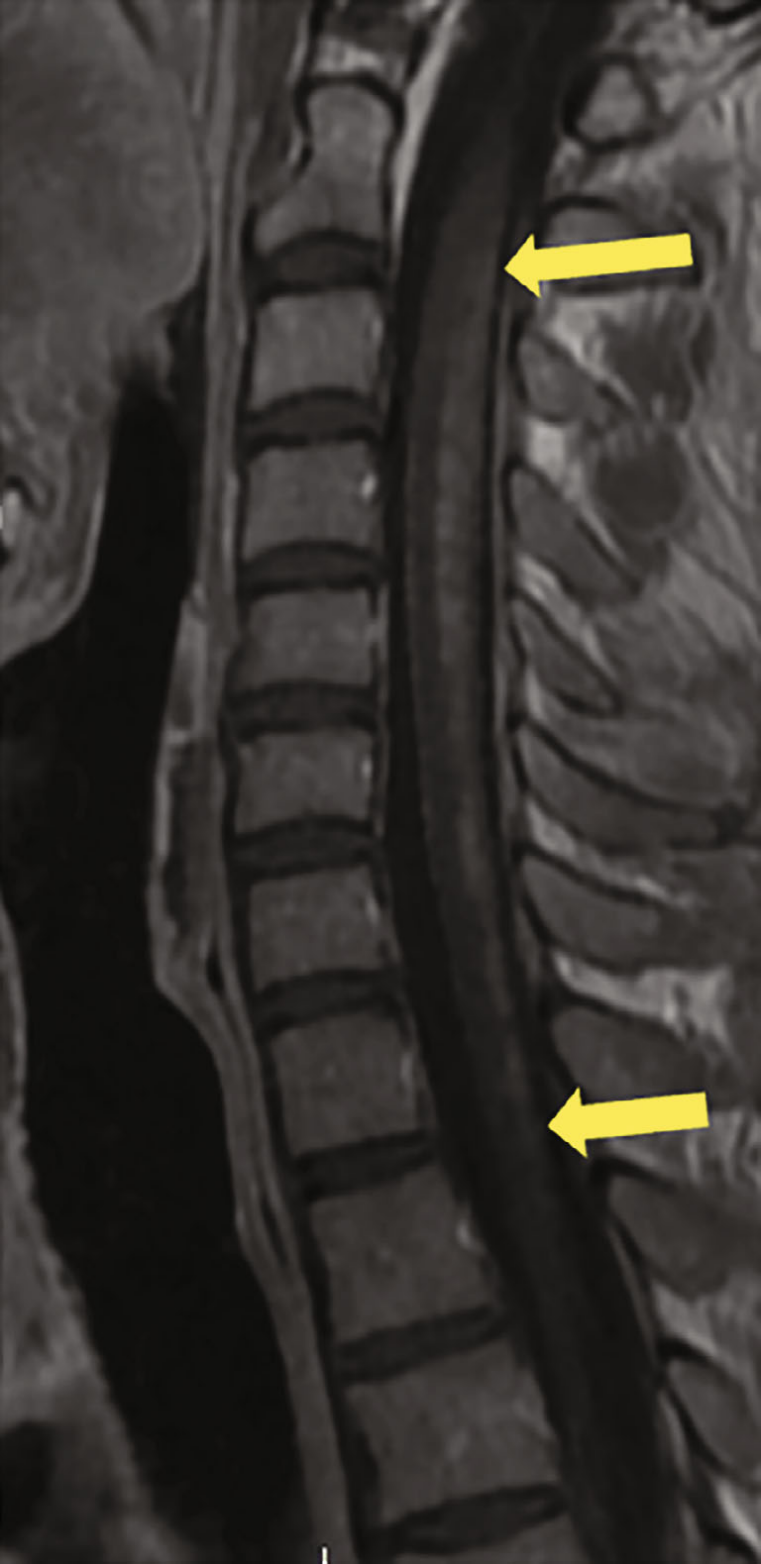
(d)

## 3. Discussion

Nitrous oxide (N_2_O), also known as “laughing gas,” is an inhalant that is used medically for short‐lasting procedural analgesia and for dental procedures [5]. It functions as an N‐methyl‐D‐aspartate antagonist with added opioid receptor activity, resulting in euphoric and analgesic effects, which have led to widespread recreational abuse [5, 6]. Persistent use of nitrous oxide may cause inactivation of vitamin B12 and subsequently lead to SCD. N_2_O‐mediated SCD causes adverse effects such as spastic paraparesis, impaired vibration and proprioception sense, limb weakness and numbness, myelopathies, and other neurological symptoms [2, 7]. These symptoms occur due to the development of lesions in the spinal cord located in the lateral corticospinal tracts and dorsal columns [4].

Diagnosis of N_2_O‐mediated SCD is made with laboratory tests that include plasma homocysteine, methylmalonic acid (MMA), and serum vitamin B12, though levels may vary or be normal. MRI scans of the cervical and thoracic spine may confirm the diagnosis and can help in eliminating alternative etiologies such as primary spinal cord malignancy. In around 50%–100% of patients with N_2_O‐mediated SCD, MRI shows dorsal column T2 hyperintensity in the cervical spinal cord [8]. Imaging classically shows a bilateral, symmetric, longitudinally extensive hyperintensity, often described as an “inverted V sign” or “rabbit ears” on axial T2‐weighted imaging [1]. Lesions may span multiple vertebral segments and predominantly involve the posterior columns, although in some advanced cases, lateral corticospinal tracts may also be involved [9]. These characteristic imaging findings, although not pathognomonic, are highly suggestive of functional B12 deficiency and are critical for early diagnosis in ambiguous clinical presentations. MRI can also help monitor treatment response, with resolution or improvement in signal abnormalities often correlating with clinical recovery [8].

Prompt recognition of nitrous oxide‐induced neurotoxicity is critical, as a delay in treatment may result in irreversible neurological deficits. Clinicians should regularly inquire about recreational drug use, particularly in patients who present with unexplained myelopathy or neuropathy, in order to avoid missed or delayed diagnosis. Nitrous oxide‐induced neurotoxicity has been increasingly reported among young populations who engage in recreational use, which underscores the need for heightened awareness and preventative measures [10]. Notably, some individuals may develop SCD even while self‐administering vitamin B12 supplements, suggesting that oral supplementation may be insufficient to counteract the inactivation of vitamin B12 by nitrous oxide [11]. Treatment of N_2_O‐mediated SCD includes abstinence from nitrous oxide alongside parenteral injections of vitamin B12 [2, 5, 7].

## 4. Conclusion

This case highlights the neurological consequences of chronic nitrous oxide abuse, specifically its role in inducing SCD of the spinal cord through functional vitamin B12 deficiency. Establishing the diagnosis is made through recognizing the clinical symptoms, a history of nitrous oxide use, and MRI findings of dorsal column hyperintensities. Early recognition of the disease and initiation of high‐dose parenteral vitamin B12 therapy can lead to clinical improvement, which highlights the importance of early intervention. Recreational nitrous oxide use is becoming increasingly prevalent, and clinicians should maintain a high index of suspicion for N_2_O‐induced neurotoxicity in patients presenting with vitamin B12 abnormalities and myelopathic symptoms.

## Consent

No written consent has been obtained from the patients as there is no patient identifiable data included in this case report.

## Conflicts of Interest

The authors declare no conflicts of interest.

## Funding

No funding was received for this manuscript.

## Data Availability

Data sharing not applicable to this article as no datasets were generated or analysed during the current study.
